# Cocaine Anæsthesia Rendered Harmless by the Addition of Trinitrine

**Published:** 1894-12

**Authors:** 


### Cocaine Anæsthesia Rendered Harmless by the Addition of Trinitrine.

The author proposes the following formula in which trinitrine is introduced, with the effect of preventing the anæmia of the brain:

| | | |
|---|---|---|
| R. | Cocaine muriate.............................. | xx centigrms. |
| | Alcohol. solution of trinitrine. 1 pr. ct. | x gtt. |
| | Distilled water.................................. | x grms. |

Each cubic centimeter contains two centigrammes of cocaine and one drop of the trinitrine solution. Gauthier has used this formula for two years with great satisfaction.—(*Revue gen. de Clin. et de Ther., No.* 37, 1893.)

